# When Citizen Science highlights alien invasive species in France: the case of Indochina mantis, *Hierodula patellifera* (Insecta, Mantodea, Mantidae)

**DOI:** 10.3897/BDJ.8.e46989

**Published:** 2020-01-07

**Authors:** Nicolas Moulin

**Affiliations:** 1 Nicolas Moulin Entomologiste, Honorary associate at MNHN, Montérolier, France Nicolas Moulin Entomologiste, Honorary associate at MNHN Montérolier France

**Keywords:** New record, praying mantis, human transport, distribution, Mediterranean area

## Abstract

**Background:**

Originally from Asia, *Hierodula patellifera* (Serville, 1839) occurs several Mediterranean countries, such as Italy. These arrivals could come from many factors: new pets or commercial human transport.

**New information:**

The presence of *Hierodula patellifera* (Serville, 1839) is here reported for the first time in France. A well settled and probably widespread population of this species is here discussed as its adaptability to the Mediterranean climate. Some considerations on the potential impacts on the local ecosystems and its future spreading in Europe as an invasive species are given.

## Introduction

In France, citizens interested in wildlife or enlightened amateurs use online applications or websites to learn more about the biodiversity surrounding them. They can also discuss their sightings on forums and social media. Since May 2018, it is possible to take part in species surveying by transmitting sightings using INPN Espèces. Experts study the photographs after several filters to validate or correct the data. INPN Espèces is the only mobile application in France that allows the discovery of all the French fauna and flora species (mainland and overseas). Amongst these groups, insects are widely represented. In parallel, entomologists use, since 1999, the forum hosted on insecte.org to discuss their sigthings and upload them into a gallery of photographs where they are validated by the community. A third widely-used website for Citizen-Science is inaturalist.org. This website, which is also available as a mobile application, has been used to collect collateral information and monitor the presence of *Hierodula tenuidentata* Saussure, 1869 in Italy, in the Po valley (Battiston et al. 2018, Battiston et al. 2019).

On 1 September 2019, a photo of a Mantodea male, located in Marseille, was put online with the app INPN Espèces and its identity discussed within the community. Sightings of alien Mantodea were then also highlighted on the forum insecte.org. The results of this research are presented here, highlighting the presence of a new Asian mantid species for France since at least 2013: *Hierodula patellifera* (Serville, 1839).

In addition, several species of mantids seem to invade Europe from Caucasus (Battiston and Massa 2008) and Asia (Battiston et al. 2017, Battiston et al. 2018, Battiston et al. 2019). The Greek islands are affected, but also the countries bordering with the Black Sea and finally Italy. The distribution of these species will be discussed, as well as their potential impact on local biodiversity. Climate is probably the most limiting factor for the distribution of the mantids in the Euro-Mediterranean area as for other insects (Logan et al. 2019). Some plant associations characterise the area: coniferous forest, deciduous forest, Mediterranean shrublands (maquis, garrigue) and the steppe (Battiston et al. 2010). Currently, macquis and garrigue seem to be the preferred habitats for these invasive species.

## Materials and methods

Recently, Citizen Science in France relies heavily on the application INPN Espèces. The sightings of insects are based on the website insecte.org and the World’s fauna and flora observations are organised on the online database inaturalist.org.

These three methods of publishing sightings in France are presented here:

INPN Espèces

A genuine tool for raising awareness and knowledge, building on the French biodiversity, INPN Espèces allows everyone to discover, from their mobile phone, the diversity of the species around us and to participate in the biodiversity inventory in France. It is the only app in France that allows us to investigate all the French Fauna and Flora species (mainland and overseas) through their characteristics, distribution or conservation status. Thanks to a data entry eased by images and geolocalisation from one’s mobile, anyone can, with one click, improve the knowledge on the surrounding wildlife.

To take part in the country’s species surveying by transmitting our records to experts, only a few clicks are needed to share the sightings. The first identification by the observer is then checked by experts of each group involved in the project.

insecte.org (‘Le Monde des insectes’)

This website is a gathering places for all insect lovers, whatever their level of knowledge, their approaches and their objectives. A discussion forum is organised according to the different orders of insects. When images in a post are validated collectively, the photos are placed in a gallery that serves as a reference library. Once a year, data are sent to the French national species inventory (INPN).

inaturalist.org

One of the world’s most popular online wildlife databases, iNaturalist helps everyone identify the plants and animals around us. It allows connection to a community of over 750,000 scientists and naturalists who can help us learn more about nature. Moreover, by recording and sharing our sightings, people will create research quality data for scientists who are working to better understand and protect nature. iNaturalist is a joint initiative by the California Academy of Sciences and the National Geographic Society.

The systematics of Mantodea used here follow the latest update (Schwarz and Roy 2019).

## Data resources

On 1 September 2019, a photograph of a Mantodea male resembling *Mantis religiosa* (Linnaeus, 1758) was published, from the 9th district of Marseille (Bouches-du-Rhône, France) (Fig. 1). The author, as an expert entomologist, was in charge of validating this photograph, amongst many others, illustrating the different species of Mantodea living in France. First, the observed specimen was considered as a species different from those known from France. The author of the photograph was contacted. Two days later, the same or another specimen returned to the observer's balcony, attracted by the light in the evening. It was photographed, captured and sent to the author.

In parallel, research was initiated on the forum of the website insecte.org, as well as on inaturalist.org. Alien Mantodea's records from the south of France were found on insecte.org. The identity of the specimens was discussed. Three discussions were highlighted: one with two adult females from Mouriès (Bouches-du-Rhône, France), one with a juvenile at Arles (Bouches-du-Rhône, France) and, finally, one with a female travelling on a boat in the Black Sea.

Specimens from Mouriès:

On 3 November 2013, a female Mantodea was observed in an olive grove. It was compared with a female *Iris oratoria* (Linnaeus, 1758) on a photo published on insecte.org. During the many exchanges between naturalists, its identity was discussed between *Sphodromantis viridis* (Forskål, 1775) and *Hierodula transcaucasica* Brunner von Wattenwyl, 1878. On 28 September 2015, a new Mantodea female was discovered in the same locality. It seemed to be the same species. It was kept and put in breeding to observe an ootheca and to photograph it. It had been stated that the specimens observed were *Hierodula transcaucasica*. The different people involved in the discussion were contacted. Detailed photographs were taken of the second captured female. The exact location of the captures was also noted. Oothecae and an old female were observed on olive trees in early November 2019 (Fig. 2).

Specimens from Arles:

On 27 July 2019, a nymph Mantodea was observed in a garden, on mint. Its identity was discussed. Macroscopic photographs were taken. On 5 September 2019, an adult specimen was found at the same place (probably the same specimen, developed to adult). It turns out that it was a female. Its identity was not fixed at the end of the discussions.

Specimen on the Black Sea:

On 22 September 2011, an adult female was photographed on a boat on the Black Sea. It was first mistakenly identified as *M. religiosa*. Discussions then orientated for the identification towards *H. transcaucasica*. A synonymy with *Hierodula tenuidentata* Saussure, 1869, is suggested on the basis of recent references.

Specimen from Marseille:

The male from Marseille was studied morphologically. It is *H. patellifera*. It was compared with a male of *H. tenuidentata* from the Greek island of Skopelos. Some morphological criteria, such as yellowish callous spots of the anterior coxae, are characteristic and can be observed on the photographed specimens. After examining the photographs of females from Mouriès and the nymph (then adult) from Arles, it is possible to identify them as *H. patellifera*.

On the other hand, the morphological criteria of the female on the boat on the Black Sea and its geographical location indicate that it is *H. tenuidentata*.

## Taxon treatments

### Hierodula patellifera

Serville, 1839

4A66F50F-FA2C-5B96-BDB4-D5106A1E3CF2

#### Materials

- **Type status:** Other material. **Occurrence:** recordedBy: Mattias Perez; individualCount: 1; sex: female; lifeStage: adult; **Location:** continent: Europe; country: France; stateProvince: Bouches-du-Rhone; county: Mouries; verbatimElevation: 30 m; verbatimLatitude: 43.69858; verbatimLongitude: 4.85217; verbatimCoordinateSystem: decimal degrees; **Identification:** identifiedBy: Nicolas Moulin; **Event:** samplingProtocol: hand catching; eventDate: 2013-11-03; eventRemarks: on olive
- **Type status:** Other material. **Occurrence:** recordedBy: Mattias Perez; individualCount: 1; sex: female; lifeStage: adult; **Location:** continent: Europe; country: France; stateProvince: Bouches-du-Rhone; county: Mouries; verbatimElevation: 30 m; verbatimLatitude: 43.69858; verbatimLongitude: 4.85217; verbatimCoordinateSystem: decimal degrees; **Identification:** identifiedBy: Nicolas Moulin; **Event:** samplingProtocol: hand catching; eventDate: 2015-09-28; eventRemarks: on olive
- **Type status:** Other material. **Occurrence:** recordedBy: Gauillaume Paulus; individualCount: 1; sex: female; lifeStage: nymph; **Location:** continent: Europe; country: France; stateProvince: Bouches-du-Rhone; county: Arles; verbatimElevation: 5 m; verbatimLatitude: 43.673112; verbatimLongitude: 4.633544; verbatimCoordinateSystem: decimal degrees; **Identification:** identifiedBy: Nicolas Moulin; **Event:** samplingProtocol: hand catching; eventDate: 2019-07-26; eventRemarks: in a garden
- **Type status:** Other material. **Occurrence:** recordedBy: Didier Aurelle; individualCount: 1; sex: male; lifeStage: adult; **Location:** continent: Europe; country: France; stateProvince: Bouches-du-Rhone; county: Marseille; verbatimElevation: 45 m; verbatimLatitude: 43.2565769; verbatimLongitude: 5.4097269; verbatimCoordinateSystem: decimal degrees; **Identification:** identifiedBy: Nicolas Moulin; **Event:** samplingProtocol: hand catching; eventDate: 2019-09-01; eventRemarks: on a balcony, 6th floor

#### Distribution

**Figure 3.**
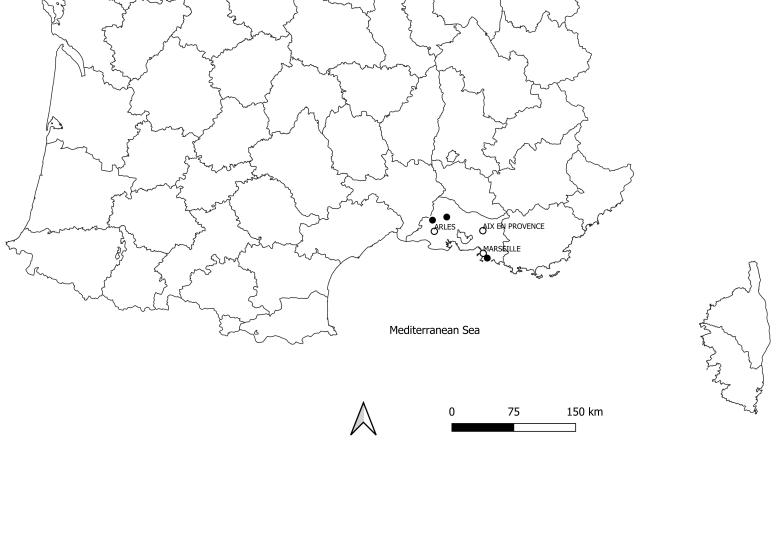
Map of all records of *Hierodula patellifera* in France.

*Hierodula patellifera*, described from Java (Audinet-Serville 1839), is a species widespread in many Asian countries: Malaysia, India, Japan, Korean Peninsula, New Guinea, Philippines, Southern China, Taiwan, Thailand, Vietnam and West Sumba (Ehrmann 2002). More recently, it has been reported from Hawaii (Ehrmann 2002, Nguyen and Maxwell 2008) and Italy (Battiston et al. 2019), presumably as the result of a human-assisted introduction. Today, it occurs in Bouches-du-Rhône, France (Fig. 3).

#### Notes

Mantodea Species File Online (Otte et al. 2019 http://Mantodea.SpeciesFile.org/) will be updated with all these observations.

## Discussion

We can state today that *Hierodula patellifera* occurs in a French department, Bouches-du-Rhône, on the basis of these records. The arrival would be prior to 2013. Several generations and a nymph have been observed. The first individuals could have arrived by Cargo from Asia or could have been voluntarily released, because it is a commonly reared pet. This would not be a continuation of colonisation with the known populations of *H. tenuidentata* in Northern Italy, in the Po Valley (Battiston, comm. pers.). The hypothesis of displacement by boat is valid because, a few years ago, a female specimen of *H. tenuidentata* was observed aboard a ship on the Black Sea. Its place of arrival in the boat could not really be located between Ukraine, Turkey, Romania, Bulgaria, Albania and the Danube Estuary. It is, however, well known that this species occurs in these countries (Battiston et al. 2017, Cianferoni et al. 2018, Heyden 2018a, Heyden 2018b).

The adults observed at Mouriès seem to develop in the olive trees and in the shrub stratum. In the Mediterranean area, one of the most representative native plants is *Olea europaea* Linne, 1753, the distribution of which represents well the Mediterranean biogeographic region (Battiston et al. 2010). In Asia, in Singapore, *H. patellifera* occurs in secondary forests, forest edges, scrubland, gardens and parks (Leong 2009).

*H. patellifera* would have no impact on native species that typically develop in the herbaceous stratum. On the other hand, the nymph observed in Arles, was on mint. If it is proven that nymphs develop in the herbaceous stratum, a problem of competition could be encountered with native species. Its life cycle in a temperate environment should overlap the life cycle of native species. Indeed *H. patellifera* is more massive than any French mantid species, as is also the case with *H. tenuidentata* in Italy (Battiston et al. 2018, Battiston et al. 2019).

The South of France is not immune to the arrival of another species of *Hierodula*, *H. tenuidentata*, coming from either the Greek islands by human transport or by a natural displacement from the Po valley, Italy (Caesar et al. 2015, Battiston et al. 2018, Cianferoni et al. 2018, Heyden 2018a, Heyden 2018b, Schwarz and Ehrmann 2018, Battiston et al. 2019).

Finally, the species of *Hierodula* that invade Southern Europe by the Mediterranean Basin are not the only ones. Another genus of Mantodea, similar to *Hierodula*, can approach France by the Iberian Peninsula and the Mediterranean islands (Cyprus, Mallorca and Sardinia) and is represented by one species: *Sphodromantis viridis* (Canyelles and Alomar 2006, Ehrmann 2011, Domenech Fernández 2018, Marabuto et al. 2014, Battiston et al. 2017, Schwarz and Ehrmann 2018, Battiston et al. 2019).

Today, thanks to citizen science, it is important and possible to monitor such invasive alien species. However, the problem of the specific attribution of these specimens through photos is important. Then, some specialists of Mantodea must be in charge to identify them.

These invasive species could destabilise existing ecological niches by creating competition and ecological pressure, like the Asian Hornet, *Vespa velutina* (Requier et al. 2018). At the moment, however, Schwarz and Ehrmann (2018) consider that competitive effects like resource depletion and intraguild predation are probably negligible. When compared to structurally comparable habitats in Africa and East Asia, Europe is depauperate in mantodeans due to past Ice Age oscillations. That is, as there is no saturation point reached yet, empty niches for newcomers are provided.

## Supplementary Material

XML Treatment for Hierodula patellifera

## Figures and Tables

**Figure 1a.**
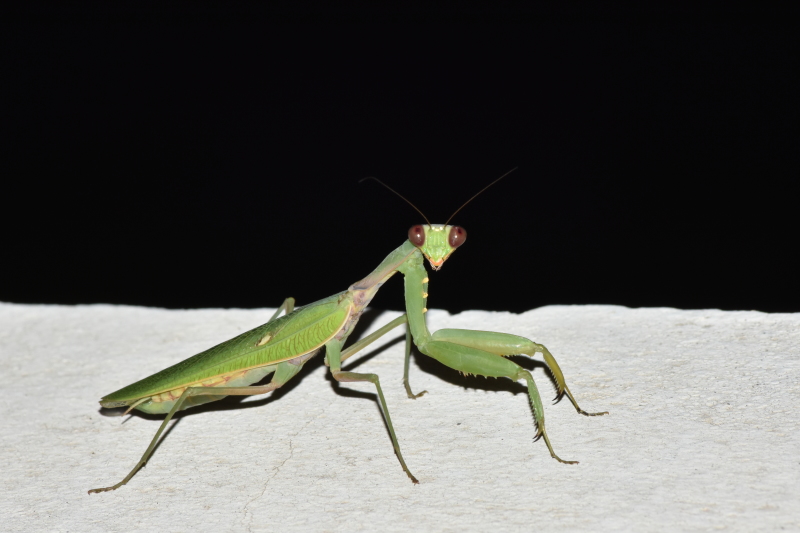
Male *Hierodula patellifera* from Marseille 9^th^ district (Bouches-du-Rhône) – credit Didier Aurelle.

**Figure 1b.**
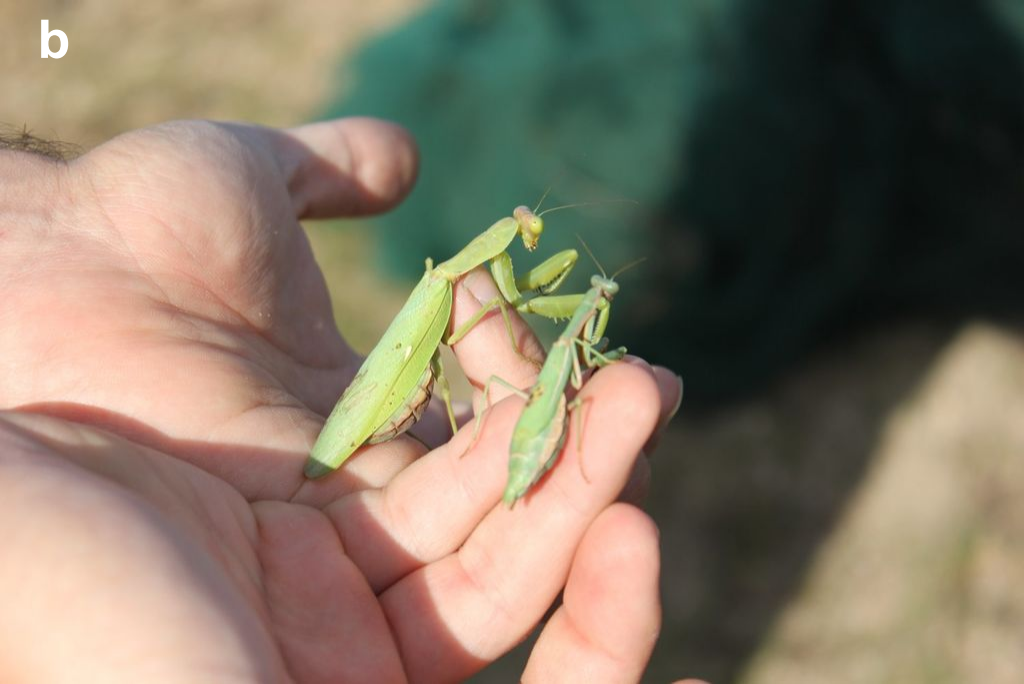
Females *Hierodula patellifera* (left) and *Iris oratoria* (right) from Mouriès (Bouches-du-Rhône) – credit Mattias Perez.

**Figure 1c.**
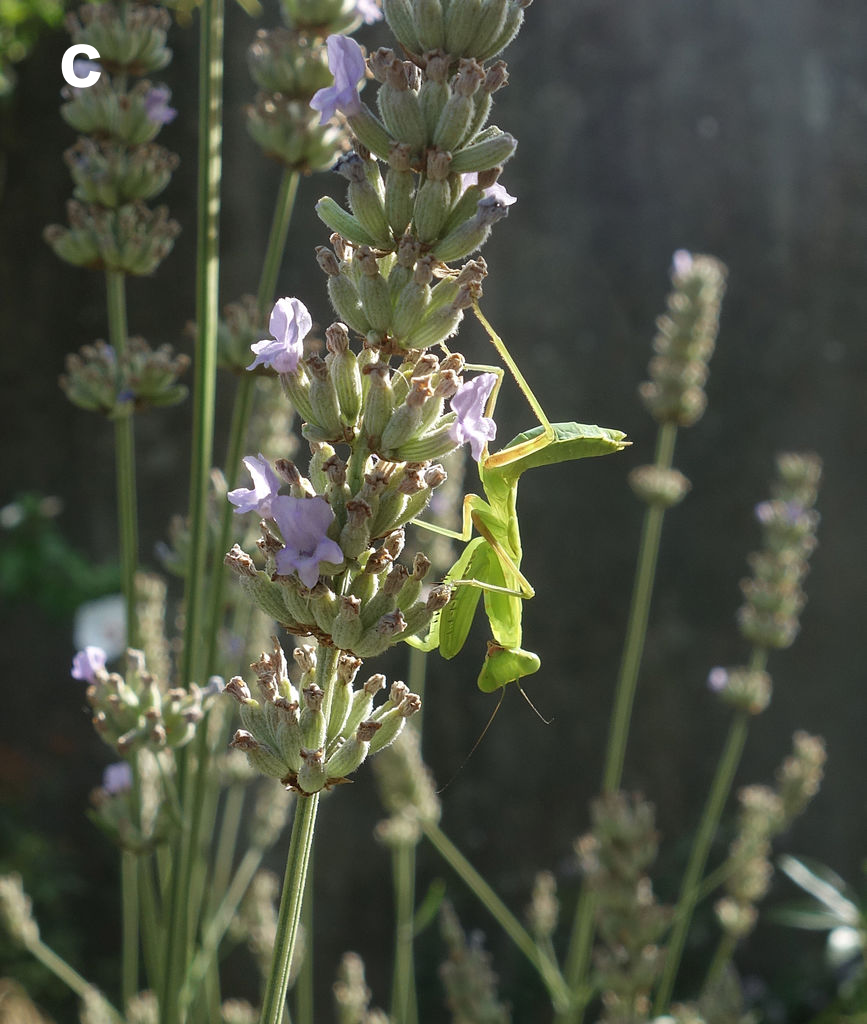
Nymph *Hierodula patellifera* from Arles (Bouches-du-Rhône) – credit Guillaume Paulus.

**Figure 1d.**
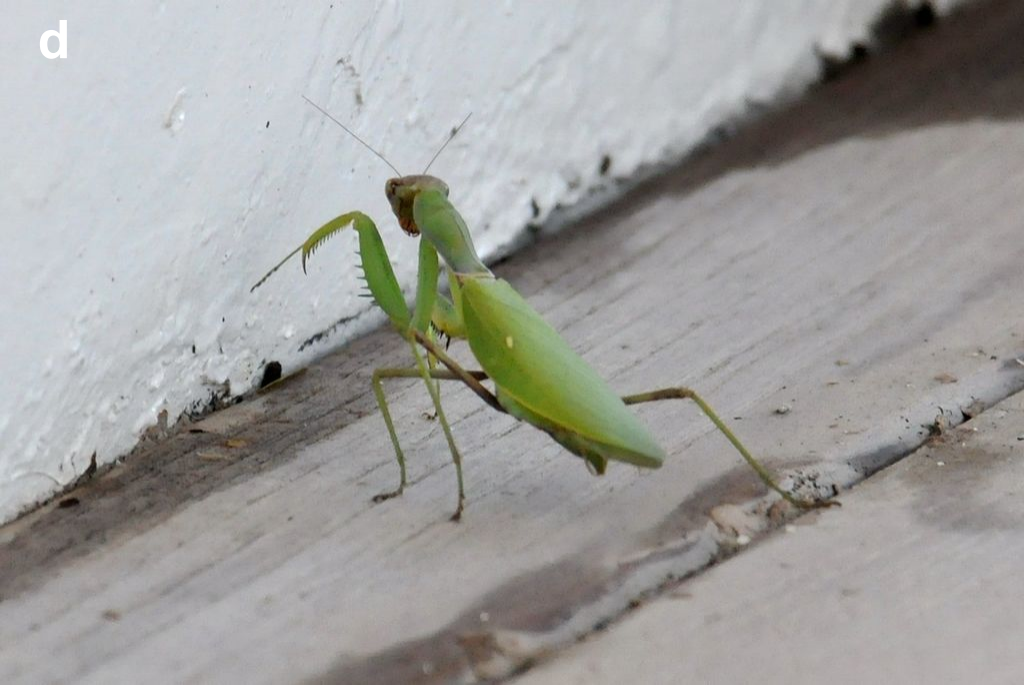
Female Hierodula cf. tenuidentata on a boat in the Black Sea, between Istanbul and Yalta, probably Nessebar, Bulgaria – credit Jean-Claude Jamoulle.

**Figure 1e.**
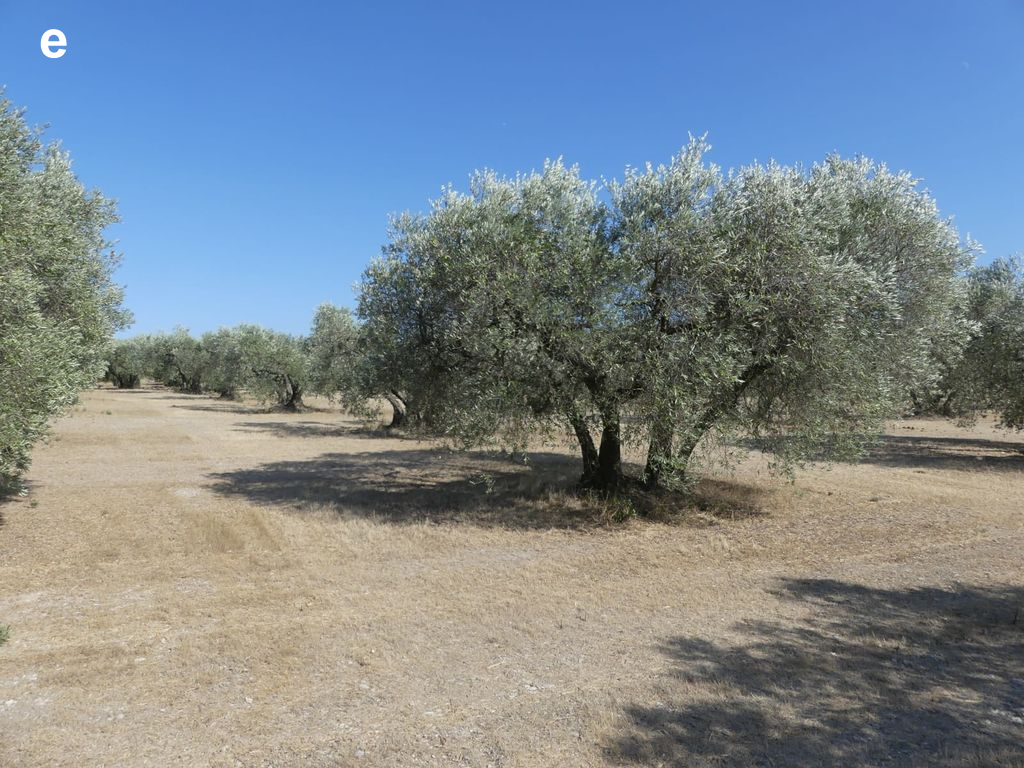
Olive grove near Mouriès (Bouches-du-Rhône) – credit Patrick Moulin.

**Figure 1f.**
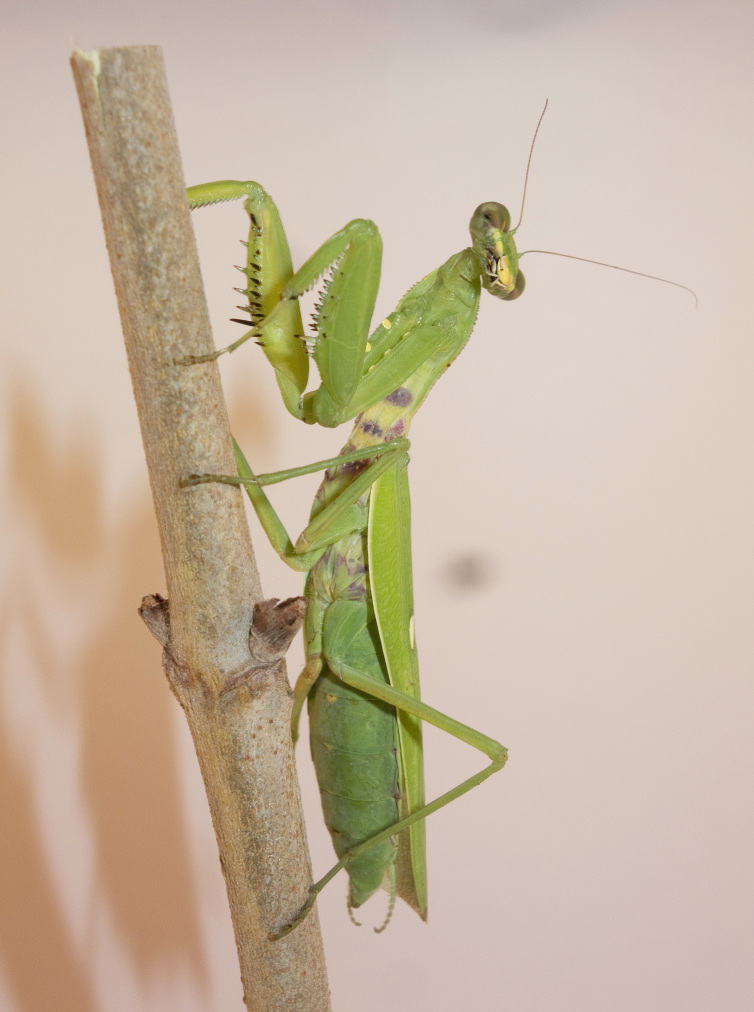
Female *Hierodula patellifera* (probably nymph of **c** which has become adult) from Arles (Bouches-du-Rhône) – credit Guillaume Paulus.

**Figure 2a.**
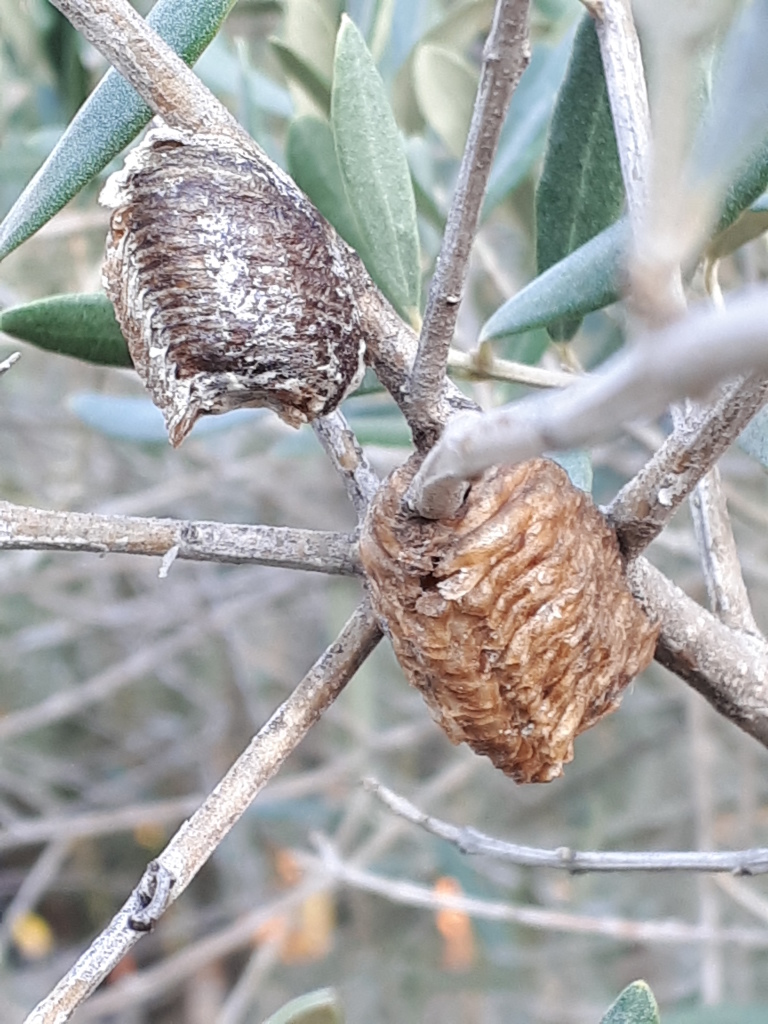
Old and recent Oothecae on an olive tree - credit Patrick Moulin

**Figure 2b.**
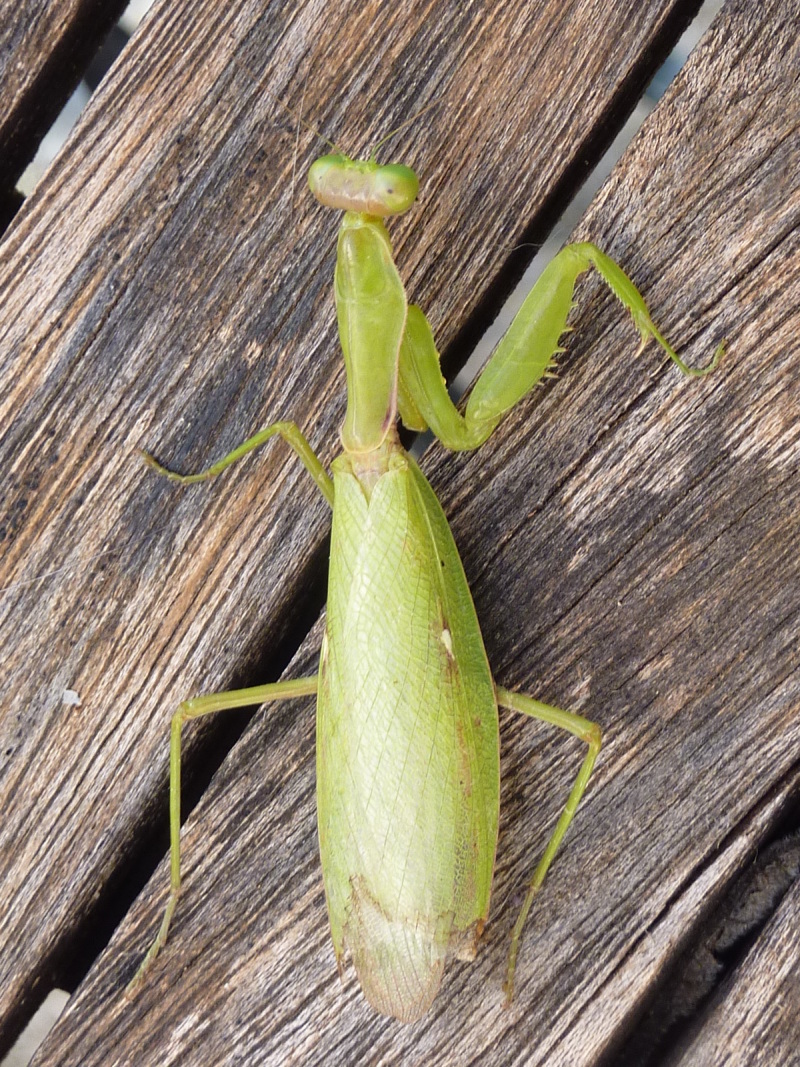
Old female at Mouriès - credit Sylvie Bucci
